# Towards Smart Home Automation Using IoT-Enabled Edge-Computing Paradigm

**DOI:** 10.3390/s21144932

**Published:** 2021-07-20

**Authors:** Hikmat Yar, Ali Shariq Imran, Zulfiqar Ahmad Khan, Muhammad Sajjad, Zenun Kastrati

**Affiliations:** 1 Digital Image Processing Laboratory, Islamia College Peshawar, Peshawar 25000, Pakistan; hikmatyar4375@gmail.com (H.Y.); muhammad.sajjad@ntnu.no (M.S.); 2 Norwegian Colour and Visual Computing Laboratory, Department of Computer Science (IDI), Norwegian University of Science and Technology (NTNU), 2815 Gjøvik, Norway; 3 Intelligent Media Laboratory, Digital Contents Research Institute, Sejong University, Seoul 143-747, Korea; zulfiqar@sju.ac.kr; 4 Department of Informatics, Linnaeus University, 351 95 Växjö, Sweden; zenun.kastrati@lnu.se

**Keywords:** smart home, home automation, cloud computing, edge computing, raspberry pi, internet of things

## Abstract

Smart home applications are ubiquitous and have gained popularity due to the overwhelming use of Internet of Things (IoT)-based technology. The revolution in technologies has made homes more convenient, efficient, and even more secure. The need for advancement in smart home technology is necessary due to the scarcity of intelligent home applications that cater to several aspects of the home simultaneously, i.e., automation, security, safety, and reducing energy consumption using less bandwidth, computation, and cost. Our research work provides a solution to these problems by deploying a smart home automation system with the applications mentioned above over a resource-constrained Raspberry Pi (RPI) device. The RPI is used as a central controlling unit, which provides a cost-effective platform for interconnecting a variety of devices and various sensors in a home via the Internet. We propose a cost-effective integrated system for smart home based on IoT and Edge-Computing paradigm. The proposed system provides remote and automatic control to home appliances, ensuring security and safety. Additionally, the proposed solution uses the edge-computing paradigm to store sensitive data in a local cloud to preserve the customer’s privacy. Moreover, visual and scalar sensor-generated data are processed and held over edge device (RPI) to reduce bandwidth, computation, and storage cost. In the comparison with state-of-the-art solutions, the proposed system is 5% faster in detecting motion, and 5 ms and 4 ms in switching relay on and off, respectively. It is also 6% more efficient than the existing solutions with respect to energy consumption.

## 1. Introduction

In the last few decades, many practitioners have focused on connecting everyday objects, including appliances, sensors, and actuators, to the Internet to enable users to control and monitor things anytime and anywhere. This revolution invented the term IoT [1] and IoE (Internet of Everything) [2], which make everyday objects more smart and able to perform complex tasks. The IoT is a global network of information for smart homes and smart cities composed of Internet-connected objects such as Web of Objects (WoOs), things, and other smart devices of the Internet [3]. The WoOs [4] represent and support the intelligent features of real-world objects as web resources, based on REST (Representational State Transfer) principles, and can be accessed through lightweight Application Programming Interface (APIs) [5,6]. The overall home devices need to communicate with each other and end-users to use smart home environment resources efficiently. 

Smart home or home automation is the process of automatically controlling different home appliances or devices and programing them to replace several human interactions for handling essential home functions. The connected sensors and devices are operated via IoT supported platform, providing connectivity and control to them worldwide. Such interconnectivity capabilities allow smart home devices to acquire real-time information from different places, improving customer safety and security [7]. Safety and security is another vital application of the smart home system. In recent decades, the increase in burglary, theft, and similar security breaches puts our lives at risk [8]. Intelligent home systems also provide smart protection to ensure human life’s safety and security by automatically monitoring activity recognition, elderly fall detection, fire detection, smoke detection, gas detection, invasion, and home surveillance. 

Besides automation and security, the smart home system also provides efficient energy management systems to effectively use electricity in our homes [9,10]. Energy management systems also play an essential role in residential electricity usage due to the current rapid evaluation in smart home technology coupled with rising inhabitants. According to the World Business Council for Sustainable Development, residential energy consumption is around 40% and 30% of commercial buildings globally [11] and it is approximated that the power consumption of commercial and residential buildings will be increased to 53% by 2035 [12]. The smart home system provides an efficient and effective mechanism for energy optimization in residential buildings. 

Many organizations have developed smart home systems such as August Smart Lock, HomeSeer, Connectify, and Notion that are costly and do not allow easy integration of sensors, actuators, and other IoT devices [13,14]. There are many application areas of smart homes, such as home automation, home security, and home energy management. Most of the available systems are aimed at a single application area [15,16,17,18]. They do not provide a single solution to manage all the smart home needs within a single framework, and many devices and systems work individually or independently from the others. The rapid growth of IoT technology [19] makes communication and collaboration with smart objects possible. Smart objects [20] and network connectivity are the essential components for intelligent security, smart home, and efficient energy management. In the network, the processors are responsible for controlling, monitoring, and communicating. Recently smart security networks used cloud computing [21], which offers unlimited computation and storage resources. However, there are some issues heavily related to cloud computing. In cloud computing sending all the collected data to the cloud at every time are very costly in terms of latency, storage, bandwidth, energy consumption, and so on. 

**Figure 1:**
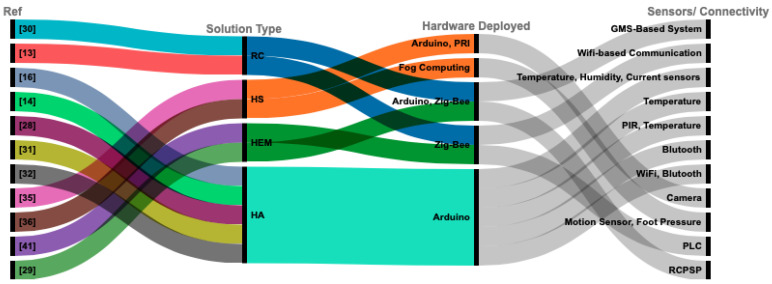
A summary of different smart home solutions including remote controlling (RC), home security (HS), home energy management (HEM) and home automation (HA).

The concept of fog and edge computing has been presented to address these issues [22,23]. In edge computing, resources such as computation, storage, etc. are made available at the network edge, close to end-devices. Placing computing resources closer to the devices generating the data reduces communication latency. Furthermore, the analysis and processing of network-intensive data are just one step away from end-devices, which reduce the demands of bandwidth on network links to distant data centers. New services can be easily enabled in processing and storing data [24,25]. Additionally, edge computing also supports mobility and geographically distributed applications which are considered the key characteristics of IoT platforms [26]. In the light of the aforementioned studies, we found that the smart home is an emerging and challenging field of research, where a significant improvement is necessary at this stage. Based on the recent literature as depicted in Figure 1 (details in Table A1), no such single interface solution exists that can efficiently cover various aspects of smart home, such as home automation, safety, security, energy consumption using edge computing without requiring third-party software and services. Therefore, we propose an automatic, secure, energy-efficient, cost-effective, and reliable solution to the smart home based on edge-computing functionality.

The main contributions of this work are:

1. Development of an integrated system for smart homes capable of controlling home appliances automatically and remotely via smartphone or PC, to provide a safe and secure environment, and to reduce energy consumption.
2. Using IoT and edge-based computation paradigms to enable large-scale data production and analysis via local systems, reducing bandwidth and computation costs.
3. A hybrid solution that takes advantage of an intelligent computational model and a secure repository for all the sensitive data generated from the system’s sensors.

The arrangement of the remaining work is as follows. In Section 2, the existing relevant techniques are discussed. In Section 3, we cover the proposed method. The results and discussion are provided in Section 4. Finally, the conclusion and future work are presented in Section 5.

## 2. Related Work

Many researchers have proposed various solutions in smart home areas such as home automation, safety, security, and energy consumption for human life facilitation. Some of these solutions are discussed in the following.

### 2.1. Home Automation, Safety, and Security

There are many approaches available for home automation, safety, and security to enhance the lifestyle of human beings. The automation part controls home appliances, such as lights, fans, and ACs [16]. Several researchers deployed a microcontroller-based home automation system in which a microcontroller is used as a server, and an Android application is used to access the system through the Internet. Usually, the Arduino board is used as a server, which is a low-cost microcontroller, and is a part of the computer, and can run only one program at a time [17,18]. This approach is further extended by [27] by integrating temperature and current (voltage and ampere) sensors with a home automation system. A wireless home automation system built on Arduino is presented in [14]. It provides two modes. A manually operated mode to control home appliances through a smartphone and a self-automated mode to automatically control home appliances through connected sensors. The hardware implementation with Matlab-GUI made the system expensive (need a PC) and required more power to run the system. A system to control and monitor home appliances, using the WLAN network based on the Arduino is presented in [28], but HTML5 supportable devices can only access this system. It also requires a PC server to run the system making it less cost-effective. A hybrid home automation system is developed in [29] in which X10 wired, and ZigBee wireless technology is used. The approach followed smart task scheduling with a heuristic for the RCPSP (resource-constrained scheduling problem). The author in [30] developed a home automation system to control and monitor home appliances through the home getaway, which is based on ZigBee, Android application, and Arduino. 

Some researchers have proposed Bluetooth enabled home automation system. For instance, in [31], the authors developed a home automation system that includes a primary controller and Bluetooth sub-controller connected to a single home device. Another Bluetooth-based home automation system which uses cell phone was presented in [2], in which home appliances are connected to the Arduino board. The cell phone and Arduino are connected through Bluetooth technology. A security filter has been used in this approach to secure the system from unauthorized access. The main disadvantage of a Bluetooth-based home automation system is that it can only be accessed in an indoor environment or within the Bluetooth range. 

A voice control home automation system has been presented in [32], which comprises two main parts, i.e., voice recognition system, and wireless system. The Android application was used for voice recognition and Bluetooth technology for connecting different modules wirelessly. In this system, the authors used three different technologies such as Bluetooth, Wi-Fi, and ZigBee. Using multiple communication devices requiring separate protocols for communicating with each component makes the system unfeasible and increases the implementation cost. Another voice-based automation system has been developed in [33], but the Open Platform Communications server makes the system costly. A Zigbee-based home automation system is proposed in [13], including home network devices and home network gateway to operate home appliances. 

Besides home automation, the safety and security is another key feature of a smart home, where several researchers proved their contribution. A home automation and home security system based on ZigBee is presented in [34], providing multi-home communication capability. However, the user controls home appliances by sending a command through SMS to the main controller. A home safety and security system is proposed in [15], in which an elderly fall and flame and gas detection mechanisms are used to protect the elderly population from any kind of hazard. The security aspect for smart homes based on the passive infrared sensor (PIR) and Arduino is proposed in [35]. In this research work, the authors used a PIR with Arduino for motion detection, and camera sensors are used with PIR for intruder detection. When the PIR sensor detects a motion, the camera sensors will be activated to capture an image. Furthermore, they used a histogram of gradient (HoG) to extract prominent features from the captured image, and these features are fed to the support vector machine (SVM) for intruder detection. If the intruder is detected, the system turns on the alarm to warn the homeowner about the activity. The experimental results demonstrated that it only takes two seconds for detecting motion, and their approach reached an accuracy of 89% for intruder detection. The system had a high misclassification rate and often triggered an alarm for normal activity. There is still room to improve the accuracy in this case by using modern mechanisms. In [36], the authors incorporate fog computing technology to analyze foot size, pressure, and movement in real time for person identification. A predictive learning-based Adaptive Neuro-Fuzzy Inference System (ANFIS) is used for intruder detection. Furthermore, in this approach, they raised an alarm in the case of any emergency in real-time situations. Their work is validated in a smart home environment database selected from an online repository called the UCI, which comprises 49,695 records. It consists of Identity-based parameters, foot size, pressure, namely weight, and movement. Superior performance is achieved by their proposed work as compared to other SoTA prediction models. Jan et al., [15] proposed a technique for detecting a person falling on the floor, flames, and leakage of any harmful gas detection. This system is aimed for elderly persons and uses an RPI-based prototype that can be easily mounted to the elder people as a safety device. In the case of any emergency, the system is responsible for sending an alarm message to their relatives along with their global position system (GPS) location.

### 2.2. Home Energy Consumption

Besides automation, safety, and security, another important aspect of the smart home is the reduction of energy costs. For residential usage, energy consumption is increasing day by day. For this purpose, a different module of a smart home has been implemented, such as controlling home lights automatically considering natural light. Numerous research works proposed that daylight can be substituted for limiting electricity consumption in commercial buildings automatically via light sensors. Therefore, there are many approaches available to decrease residential energy usage [37,38]. Smart home technologies include ICT, sensors, and network capability to automatically switch home appliances through a smartphone application, touch screen, or voice [39]. Smart meters and instruments provide better prospects for the user to efficiently manage and control their home electricity [40]. Han et al., in [41], presented a ZigBee-based energy management technique that measures the usage of energy by home appliances (electrical, electronic devices). They also used a power line communication (PLC)-based approach for the measurement of energy generation. For smart homes, Anvari et al. [42] developed a multi-objective mixed integer nonlinear programming model for optimal energy use. The result showed that the algorithm not only reduced utility bills and domestic energy usage but also provided optimal task scheduling and a thermal comfort zone for the residents. In [43], the researchers provided an efficient mechanism to control the energy consumption of two different climate regimes such as Algiers and Stuttgart, cities in Algeria and Germany. The solution was aimed at a single-family house, but it was not cost-effective due to the implementation cost.

### 2.3. IoT Platforms for Smart Home

The network is an important part of smart objects connectivity. Smart objects include controllers, sensors, actuators, and different processors, which are used to control, monitor, and communicate with each other in the network [20]. Smart home takes advantage of cloud computing [44], but there are significant deficiencies in cloud computing including latency and response time. For this purpose, Li et al. proposed a technique in [45] to overcome cloud computing limitations. They studied the problem of data latency and response time of the smart object (used in smart homes and smart cities) in cloud computing and decided to switch from cloud to fog computing, which enabled the real-time interaction of the smart object, overcoming latency and data volume and speed issues. Scalability is another issue in cloud computing as discussed by Faruque et al. [46]. Fog computing provides a better energy management strategy. Adaptability, Scalability, and open-source hardware/software included in the fog computing paradigm facilitate the user to reduce the implementation cost, time, and energy consumption with customized control-as-service. Perera et al. [47] studied resource wastage issues in cloud computing and network storage. However, a new fog computing paradigm that has limited computational capabilities at the edge cannot address this challenge alone. To address this challenge, both paradigms need to collectively build supportable IoT infrastructure for smart cities. Fog computing faces new privacy and security issues. In the perspective of fog computing, Yi et al. [48] discussed several security issues including data storage security, computation security, and security of the network, and highlighted some other issues regarding data privacy, user privacy, and privacy of the location. Most fog computing applications in IoT only collect data from homogeneous IoT devices but cannot collect data from hybrid IoT devices into one real IoT application. Lu et al. [49] introduced a lightweight privacy-preserving aggregation scheme of data for fog computing to enhance the usage of fog computing in IoT applications. The lightweight privacy-preserving data aggregation (LPDA) employs different privacy techniques, Chinese remainder theorem, homomorphic Paillier encryption, and one-way hash chain technique. Amadeo et al. [50] conducted a study where they highlighted the benefits of fog computing over cloud computing. They rely on Information-Centric Networking to control and monitor the smart home environment and presented a reference architecture as proof of concept. 

Edge computing has recently gained a lot of attention in which the data are processed over the edge overcoming dependency, latency, security, and data privacy issues. It is an ideal paradigm for designing efficient home solutions comprising various IoT devices. Our proposed system takes advantage of this paradigm. It has shown to significantly improve the response time and the latency issues operating multiple home appliances.

Figure 1 shows the solution’s type of hardware deployed, sensors, and communication protocol used in various research studies. The specifications are also tabulated in Table A1 for an easy comprehension.

Our proposed solution differs from the existing works in four different aspects: First, our solution uses RPI (system-on-chip device) as a central controller that can provide remotely controlling, sensors-based automation, security, safety and reduce energy consumption at a single platform. Second, all the sensors are directly connected to the RPI with both wired and wireless mediums, and Wi-Fi is also used for Internet connectivity to control the entire system from all over the globe. Third, to reduce cloud computing dependency, we applied a 2 TB storage device named My-Personal cloud device at the network edge for the storage of all sensors data which can be further processed in the future for different prediction purposes such as weekly, monthly, or yearly temperature prediction, weather prediction, peak hours load estimation for efficient energy management, etc. Fourth, our proposed solution can be easily integrated with the existing architecture (main electric board).

## 3. Proposed Framework

**Figure 2:**
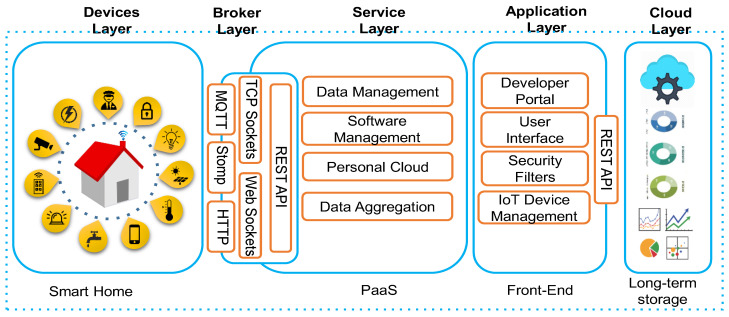
The proposed framework consists of five layers such as devices, broker, service, application, and cloud layers.

Home automation, security, safety, privacy, and energy consumption are emerging technology fields and can be implemented with different microcontrollers and processors, such as Desktop PC, RPI, Arduino, etc. All these microcontroller and processors have their pros and cons, but RPI is the one that is more efficient as compared to other units because of its unique features. RPI is based on ARM technology which is used on the board, reduces computation cost, and power consumption. RPI is System-On-Chip (SoC) device weighing 50 gm and operates on a 5 V, 700 mA power rating. The RPI version 3 model B board is used in the proposed system which consists of a Quad-Core 1.2 GHz Broadcom processor with 1 GB RAM. It has an SD card slot used for booting operating systems such as Raspbian, Pidora, and Raspbm. It has four USB2.0 ports to connect to the peripherals such as mouse, keyboard, and Wi-Fi adapter. RPI is used as the main controller in the proposed system. All the sensors, actuators, and appliances are connected and controlled by RPI. This system provides automation, security, safety, privacy, and reduces energy. We used a 5-layer architecture for easy understanding and delivering the concept of our proposed work. In this regard, we were inspired by the recent work presented in [15,50], in which the authors used a layer-by-layer approach for delivering their concept and the main motive of their work. The proposed innovative home solution (Demo: https://www.youtube.com/watch?v=XDcYgB774C0&t=12s, accessed on 17 July 2021) is based on a five-layer framework, as shown in Figure 2, where each layer of the framework is discussed in the following sections. 

### 3.1. Device Layer

The devices layer or input layer is the first layer of the proposed framework where different kinds of scalar and visual sensors are used for automation, security, safety, and to reduce energy consumption in the smart home. This layer integrates environmental sensors into the smart home system to collect data from the surrounding environment. Scalar sensors such as fingerprint are used for door authentication. PIR motion sensor is used to detect motion in the room to control the lighting system. The thermostat sensor measures the room’s temperature to control the heating and air condition system. If the temperature of the room is higher than a specified threshold degree and motion is also true, the AC turns ON automatically; otherwise, the AC will turn OFF. Photoresistor sensors are used to measure sunlight intensity. If the sunlight is lower than a specific threshold, this will automatically turn ON the outdoor lighting system of the house and vice-versa. Water measurement sensors are used to find the water level in the water tank. If the water level is less than the specified threshold, it will automatically start the water motor, and when the water tank is full, the water motor will be turned OFF automatically.

Besides this, we used gas and smoke detection sensors to detect LPG/smoke leakage in rooms and the kitchen. In contrast, flame sensors are used to detect fire in the room. If gas, smoke, or fire is detected, the siren will automatically turn on, and an SMS alert is sent to the authorized person. The visual sensor is used to provide live streaming to the homeowner on smartphones/PC to enhance home security, and the live data will be protected from unauthorized access using a proper authentication mechanism to ensure system privacy.

### 3.2. Broker Layer

**Figure 3:**
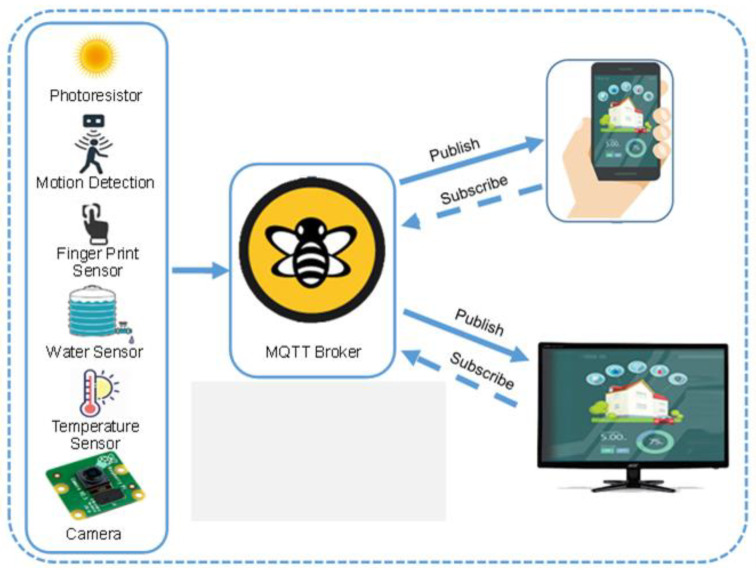
Conceptual representation of Broker layer, where MQTT broker is used to transmitting sensor data to the end-user on their smartphones or PCs.

The broker layer is responsible for transmitting data and commands from different sensors to a service layer through Message Queuing Telemetry Transport (MQTT) protocol. The working mechanism of MQTT protocols is shown in Figure 3. The broker layer is consisting of REST, Transmission Control Protocol (TCP) sockets, web sockets, Hypertext Transfer Protocol (HTTP), Simple (or Streaming) Text Oriented Message Protocol (STOMP), and MQTT protocol. RESTful web services are based on a Representational State Transfer (REST) architecture [51]. REST uses the web as an application platform and fully leverages all the features inherent to HTTP, such as browser access, scalability and caching authentication, and encryption. 

Sockets provide bidirectional communication, which is based on TCP technology. The web socket protocol is introduced in HTML5 [10]. Besides these essential communication approaches, a publish-subscribe model is introduced, which employs communication primitives from the above-mentioned protocols. In the publish-subscribe approach, a server component called broker is used to transmit the messages between publisher and subscriber. It guarantees QoS, provides similar functions and persisting messages. Many protocols are used in various IoT infrastructures, but MQTT is the most popular OASIS standard messaging protocol for the Internet of Things (IoT) due to its low-bandwidth requirements, reliability, and lightweight messaging protocol in the Internet world. MQTT is designed as an extremely lightweight publish/subscribe messaging transport that is ideal for connecting remote devices with a small code footprint and minimal network bandwidth. Every message is published to an address, known as a topic [52]. Clients can subscribe to multiple topics and receive each message published to that topic.

### 3.3. Service Layer

This layer is responsible for receiving data from the broker layer in which RPI is used as a platform or a service. We configure the Node-RED dashboard in the service layer. This cross-layer utility provides services to other layers. In the Node-RED, subscribers receive messages for given topics they subscribed to via Node-RED, which fully implements these publish/subscribe models. The Node-RED contains the nodes which can transmit the messages. A node of Node-RED-Flow (a flow is represented as a tab within the editor workspace and is primarily concerned with organizing nodes) includes JSON, JavaScript NodeJS, and HTML files. The node provides a drag-and-drop visual web-based editor, which is interconnected with other nodes for information sharing [6]. The flow editor is accessed through a web browser and navigates to http://ipaddress:1880 (accessed on 10 July 2021) The Node-RED provides input, output, function, and social nodes. We configured several flows such as smart home on/off switches, environmental sensors, room safety sensors, energy consumption, etc., in the Node-RED to control and monitor the surrounding environment. In these flows, we can turn on/off our devices, check the status of different sensors, and monitor the surrounding environments. Node-Red provides easy integration to smart home objects and quick support for scalability, which requires a simple drag and drop of a node in the Node-RED flow to make functional a new device, for instance, by assigning a GPIO pin. Furthermore, the service layer provides data management, software management, personal cloud, and data aggregation. For data storage, we used two types of storage, which are cloud storage and device storage called My-Personal cloud device. My-Personal cloud device is an easy-to-use personal storage device that plugs directly into a Wi-Fi-router at home so one can save all their digital content in one central place while cloud storage is provided by cloud computing. Cloud computing is the transmission of computing services, i.e., software, servers, networking, etc., on the Internet [12]. Cloud computing provides storage, information access, computer resources, among others, over the Internet. Cloud Deployment models typically consist of IaaS, SaaS, and PaaS [5]. The service layer provides four basic features, which are data management, software management, personal cloud, and data aggregation. All these features are discussed in detail in the following.

#### 3.3.1. Data Management

The data generated by different sensors are managed through the same platform (RPI). The temperature sensor, motion sensor, and humidity sensor sense the data continuously. The RPI is programmed to manage these sensors’ data, for instance, to perform a specific task if the sensor activates, i.e., turn ON/OFF the light and store the data into MySQL database. The data management layer works as middleware between sensor-generated data and user application. The application is granted access to sensor data which manages it and passes it on to other layers if necessary.

#### 3.3.2. Software Management

The software management layer provides a platform for data collection where different REST APIs are used to establish communication with the sensors. The sensors, along with other management software, can access the data through REST API. Data Serialization technologies enable us to use REST API for easy data sharing and application management. 

#### 3.3.3. Personal Cloud

Cloud platform enables us to store data for long-term analysis and computation. The cloud platform can also be used to compute and analyze a large amount of data generated by scalar sensors. In this study, we used a personal cloud solution to store scalar sensors and visual sensor data in the personal cloud provided by Western Digital. It has a capacity of 2 TB, which can be extended further. The data stored in the My-Personal cloud can be accessed from different platforms including, the web, Android, and others.

#### 3.3.4. Data Aggregation

To improve latency problems in the cloud, the data are aggregated before storage in the My-Personal cloud device. The type of aggregation depends on the sensor. For instance, the temperature data are generated every hour, but the generated values are averaged, and only the mean value is stored in the cloud.

### 3.4. Application Layer

This layer, the developer portal is used to develop front-end applications, which is used to manage IoT devices via a user-friendly interface. This layer implements a user dashboard to view and control various devices via Node-RED. The end-user application is supposed to have two main functions, including showing sensors data graphically and controlling electronic devices automatically when the surrounding environment changes. 

**Figure 4:**
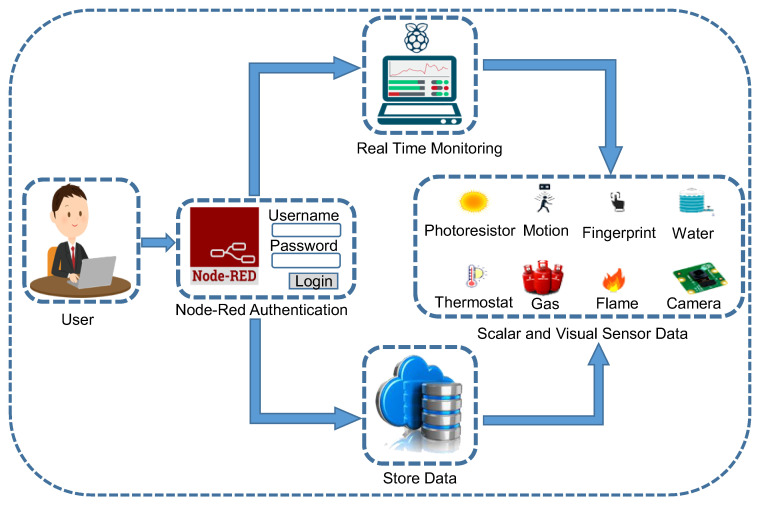
Application layer workflow, where users can sign in through their username and password for real-time data-monitoring of different sensors.

The front-end application is tested to make sure both remote and automatic electronic device controllers function as desired. The environmental changes are analyzed by the Node-RED node and tested how electrical devices react to those changes. The behaviors of the controller must meet the required expectations and respond to the surrounding environmental change without delay. Additionally, the remote controller also needs to respond instantly to the right device. Then applications must be a responsive design, which is a standard for most of the current web-based applications in the market. Particularly in this system, the web-based application is used for users who usually want to access their home controlling system from a smartphone. In the application layer, the users are responsible for authentication, as shown in Figure 4 for connecting a smartphone with a central controller through any web. The authentication process has been implemented to protect the front-end application from unauthorized access.

### 3.5. Cloud Layer

In the device layer, the proliferation of actuators and sensors generates a huge amount of raw data in a home environment that can be used to extract relevant and useful information and relationships. In most cases, there is limited storage capacity for storing all the raw data. For this purpose, a centralized storage device is necessary to extract useful and relevant information for home and community services planning. In this research work, we take advantage of cloud computing and My-Personal cloud device to store home data into an appropriate DBMS, and then at the end of the epoch, we calculate the average of each sensor data and then send it to the cloud server for long-term storage which helps for future analysis.

## 4. Result and Discussion

**Figure 5:**
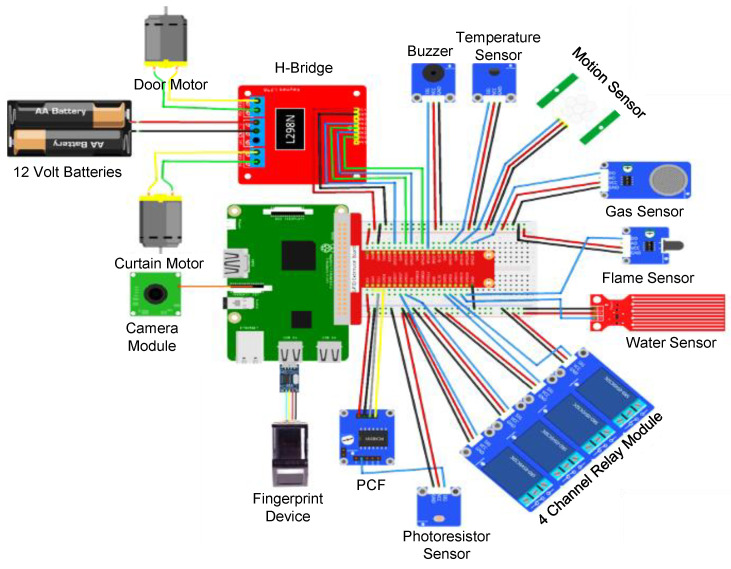
Connectivity of each module with RPI. The wires in red indicate voltage connectivity, black ones show the ground connectivity, and the wires in blue indicate signal line or data transmission line.

In this study, we propose a novel solution for home automation using an edge-computing paradigm coupled with a powerful IoT-based platform for sensor connectivity. The main theme of this work is to provide a fully automated solution that can be controlled from a centralized system with minimum cost and energy. In the proposed system, RPI is used as a central controller. All the sensors/devices are connected to it. RPI models have the processor, a system on a chip (SoC), four USB 2.0 ports, High-Definition Multimedia Interface (HDMI) port, Support for CSI (Camera Serial Interface), and GPIO (General Purpose Input and Output) pins. The proposed system has been implemented with 3rd generation RPI 3 Model B, which consists of 40 pins arranged in two rows containing 20 pins in each row. Python 3.5 programing language which provides ease of scalability, code implementation, and integration of different sensors is used. This solution can be easily implemented in different environments by attaching more sensors by just selecting the GPIO pin and a simple modification will be needed in the code. Figure 5 illustrates the connectivity of different devices with RPI, in which the red wires show voltage supply, black wires show the ground voltage connectivity, and the blue wires indicate the connection of I/O pins of different sensors with RPi GPIOs. All these devices are connected to GPIO pins except the camera, which is connected to the default camera port, and the fingerprint device, which is connected to the USB port through a TTL USB connector. This system enhances automation, security and reduces energy consumption without affecting the accuracy and performance of the system.

**Figure 6:**
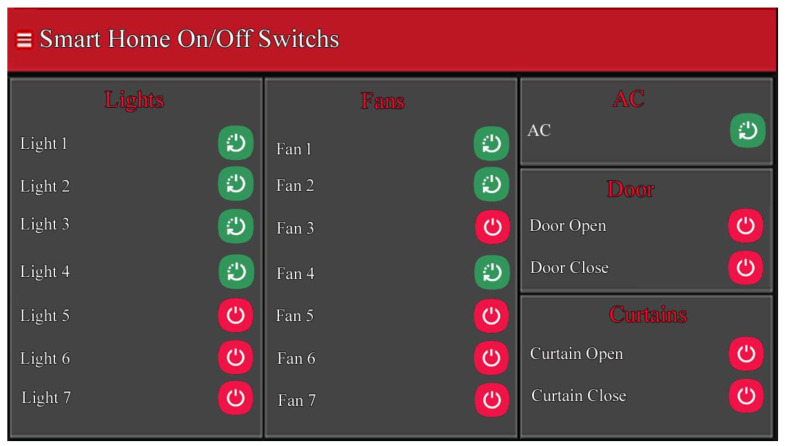
The interface to control home appliances where the red color buttons indicate that the appliances are off, and the green indicates the on status.

The devices and sensors are interfaced with RPI through wired relays and are controlled from a web-based interface through the Internet, as shown in Figure 6.

### 4.1. Controlling of Home Appliances Automatically

**Figure 7:**
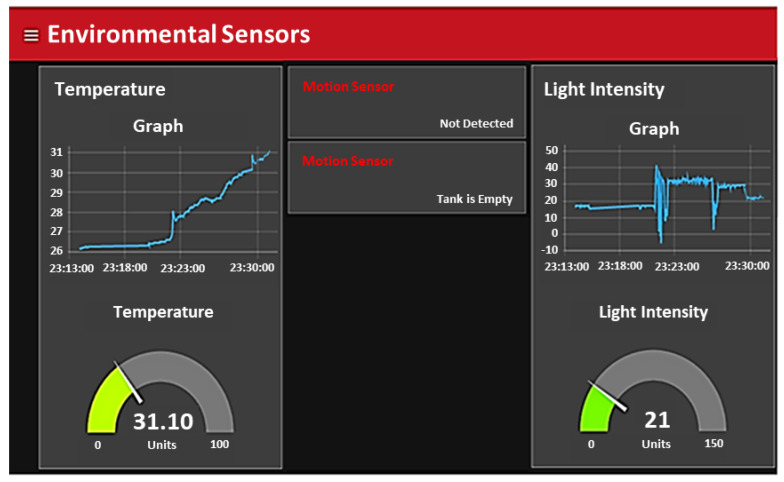
The status of environmental sensors that are used in the home for home automation.

In this section, another aspect of a smart home is discussed, i.e., home automation, where the lighting system is controlled through a PIR motion sensor, air condition system is controlled with the help of temperature sensors, and water measurement sensors are used in a water tank to automatically control water motor, streetlight automation using photoresistor sensors. In Figure 7, we illustrate the graph, gauge, and status of the environmental sensors.

Some home devices such as room lights, streetlights, AC, and water motors are automatically switching ON or switches OFF based on environmental changes. The measurement of this environmental change used a different kind of scalar sensors, as discussed in Section 3.1 in the device layer. The owner of the home can also check the current status of the home in the form of different graphs and gauges which are designed to provide a user-friendly interface, as shown in Figure 7. The temperature value of a home is presented with a gauge to display the current temperature value, and the graph of the temperature shows that the temperature values of the last few hours/days. The light intensity value is also presented in the form of a graph and gauge-like temperature. A simple text for motion and water measurement sensors is used to show the status of motion and water level in the water tank. These graphs and gauges are designed to provide a user-friendly environment to check the current condition of the home, and the system is responsible for taking appropriate action based on these sensors data, i.e., turning on and off room AC, lights, fans, water motor, etc. 

### 4.2. Home Safety

**Figure 8:**
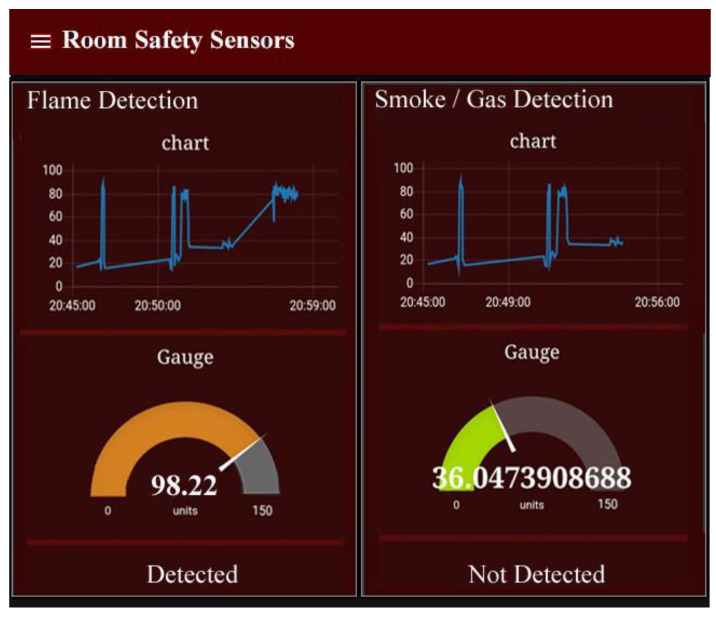
Dashboard of safety sensors in terms of graphs and gauges that are used for real-time monitoring to protect the property and human life from harm or damage.

We used a gas and flame sensor in the room, which is responsible for the monitoring of gas leakage or any open flame in the room to protect humans and their property from damage. In this case, if any open gas or flame is detected, it will automatically turn on the alarm system and sent a notification alert to the owner of the home. In Figure 8, we can see the current status of the gas and flame sensors, check its threshold level through charts and graphs such as temperature and shows the status of gas and flame whether it is detected or not. For this purpose, we set the threshold value near 150. If the threshold value is less than the specified level, it means there are no open flames and neither gas leakage. The experiment has been done by igniting a lighter and releasing the LPG gas to check whether the system works properly or not.

**Figure 9:**
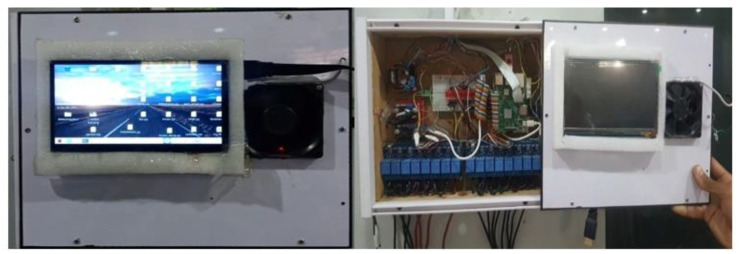
The main electric board of our proposed system.

As is shown in Figure 9, RPI is used as a central controller, the relay is used to switch on or off different devices, a breadboard is used to connect all the devices with a central controller, the fan is used for system cooling, and RPI screen is used to display information. Another key feature of the system is that the system controls home appliances automatically, which essentially means that the system takes decisions based on environmental changes. The sensors read the environment and send the relative information to be processed later. Once the decision is made, the RPI triggers the relays according to data received from the sensors and stores them in a database for further analysis. 

### 4.3. The Fingerprint-Based Door Authentication System

**Figure 10:**
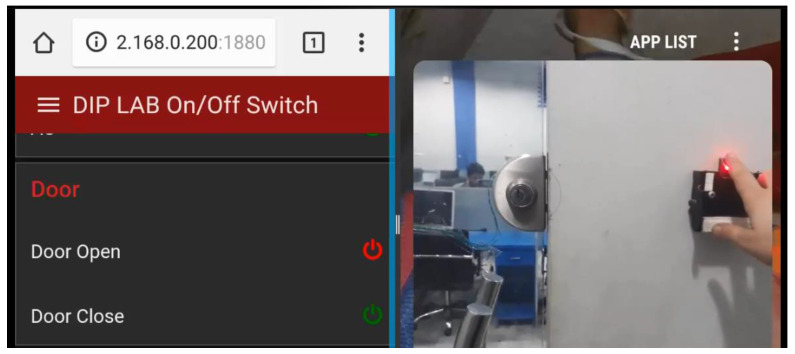
The door status using a Fingerprint-based authentication system.

In a home security system, a fingerprint-based authentication system is considered one of the most secure mechanisms [53]. A fingerprint-based authentication system has been placed at the entry point where the door is opened only for home members who are already registered in the database. The main purpose of fingerprint-based authentication is to protect the home from unauthorized access. Figure 10 shows fingerprint authentication for the door lock.

### 4.4. Online Streaming

Another type of security sensor is a visual sensor (camera), which provides online streaming to the owner of the home. The homeowner can watch online streaming on a smartphone/PC through a webpage for home monitoring.

### 4.5. Energy Consumption of the Main Controller

**Figure 11:**
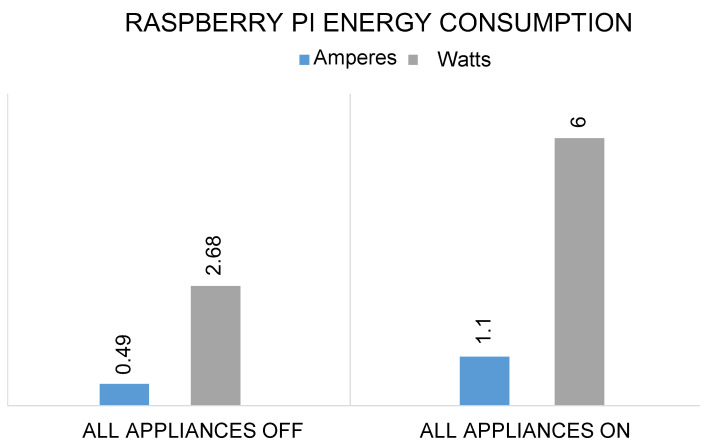
Total energy consumption of RPI in terms of amperes, and watts when all the appliances are turned ON.

**Figure 12:**
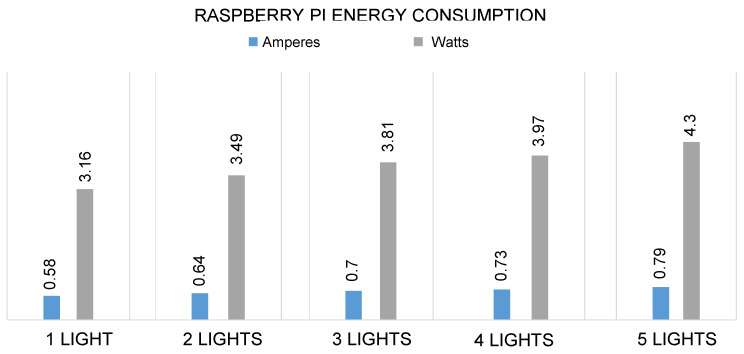
Energy consumption of RPI in terms of amperes, and watts to turn lights ON.

**Figure 13:**
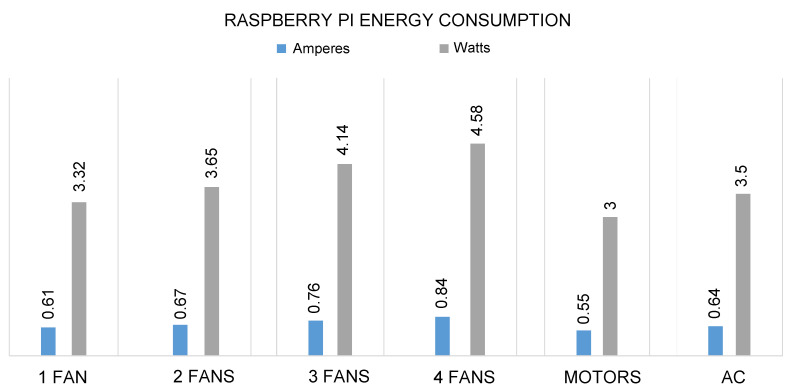
Energy consumption of RPI in terms of amperes, and watts to turn ON fans, motors, and AC.

The proposed system runs on a very low voltage and consumes much less energy to perform each operation, such as turning ON the lights, fans, ACs, and other devices. The RPI consumes 0.49 ampere, 5.45 volts, and 2.68 watts without operating all these appliances and devices in the proposed system. Similarly, the system consumes 1.1 amperes, 5.45 volts, and 6.0 watts when all the appliances and devices are operated as shown in Figure 11. Likewise, the energy consumption of lights, fans, AC, and DC motors can also be seen in Figure 12 and Figure 13. In the proposed work, RPI consumes similar voltages of 4.45 volts for each object, but the amperes and watts are different. 

Figure 13 shows the energy consumption of the system to turn fans ON, and we can see that a single fan is consuming marginally higher energy as compared to a single light. Similarly, when we increase the number of appliances, then the system consumes a little bit more energy.

### 4.6. Energy Consumption of Home Appliances

The platform of a smart home energy management system has been designed to work as a dashboard to simulate a household environment to manage, control, and monitor various household appliances. For example, customers can monitor appliance’s statuses, such as room temperature, lighting system, heating, cooling, and total power consumption. Two different scenarios are used to compare the results to illustrate the performance of the smart home energy management system in terms of managing and controlling household appliances.

**Figure 14:**
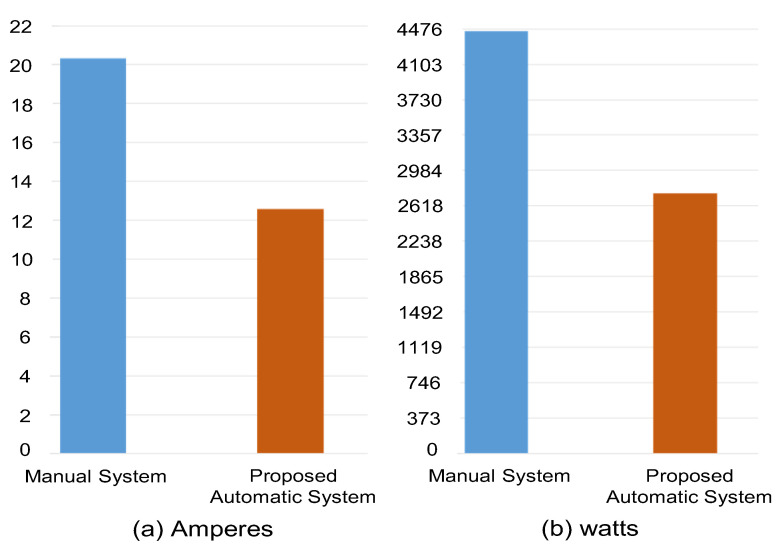
The average energy consumption of different home appliances in terms of ampere and watt used by the manual operated system and the proposed automatic one.

In the first scenario, the energy consumption of the manually operated system for a week is measured. In this case, the main concern is to measure how much energy is consumed by home appliances. In this scenario, there is no such mechanism available to reduce energy consumption at home. The average energy consumption per day using the manually operated system, in terms of voltage is 219.00 volts, the ampere is 20.33, and watt is 4452.05, as shown in Figure 14.

In the second scenario, the energy consumption of the proposed system for the second week is measured. For this purpose, a sensor-based automatic approach is used for each appliance to help in designing optimal scheduling for the smart home. However, the main concern is to use environmental sensors that help in reducing energy consumption. With the help of environmental sensors, appliances are switching off automatically if they are operating without any significance to reduce energy consumption. The average energy consumption of a week using the proposed automatic system is: 217.8 volts, 12.58 amperes, and 2739.84 watts, as shown in Figure 14.

Using automation, the same appliances consume less electricity as compared to the manually operated system. Figure 14 shows a clear difference between the manually operated system and the proposed automatic one. Apart from the optimization of energy consumption, automation offers additional benefits such as reducing/eliminating human intervention, saving time, and maximizing use of day light when possible, to name just a few. 

### 4.7. Comparison with the State-of-the-Art Solutions

In this section, we show the comparison results of our proposed system with state-of-the-art solutions concerning energy consumption and response time.

#### 4.7.1. Energy Consumption

The energy consumption aspect of the proposed system is compared with the state-of-the-art system presented in [43], which uses three different scenarios: (1) baseline model (manually operated), (2) low cost, and (3) extended for Algiers and Stuttgart, the cities of Algeria and Germany. In Table 1, it can be seen that our proposed system is deployed in Peshawar, the city of Pakistan. These three cities have different climate regimes, where the manually operated home for Algiers and Stuttgart consume similar energy according to [43], but due to the climate regimes of Peshawar, it consumed less energy, as shown in Table 1. Therefore, the table clearly shows that our proposed automatic system outperforms all other solutions by consuming less energy as compared to both scenarios of Algiers and Stuttgart cities.

#### 4.7.2. Response Time

The proposed system is compared with relevant existing smart home approaches such as ZiWi-Fog, ZiWi-cloud, and XBee, in terms of response time. The results summarized in Table 2 demonstrate that the response time of our proposed system is less than other compared methods. The response time of the proposed system is calculated by switching ON/OFF the home appliances. As a use case, we have considered light, fan, and AC, which is turned ON based on motion detection and temperature. The response time of the proposed method to turn ON/OFF home appliances considering motion detection is 15 ms for both light and fan. The average response time for turning on and off relay was 13 ms and 12 ms, respectively. The response time in [54] using fog computing is 20 ms when motion is detected to switch ON an appliance while relay switches on and off were noted as 18 ms and 16 ms, respectively. The proposed method responds 5% faster than ZiWi-Fog in motion detection. However, 5 ms and 4 ms are response times to switching relay on and off, respectively. To further evaluate the proposed system, we compared it with cloud computing methods that use cloud services. In ZiWi-cloud, a telegram [54] is used to transmit messages with each relay communication. The results demonstrate that the average time of receiving messages on a mobile phone or PC making push notifications was 2.735 s, which is quite high compared to our proposed method. Considering the IFTTT scenario, a rule is configured to check motion detection or measure temperature every 30 min. If the receiving time is too low, it switches ON a light bulb. The response time using the IFTTT service is 51.388 s. To further investigate, the method using ZigBee is also compared with the proposed system that also applies it to control home appliances. The response time in [55] was 2 s using ZigBee to switch ON/OFF appliances.

The proposed system is efficient in terms of response time compared to the SoTA methods. In the SoTA solutions, the authors used ZigBee, Node-MCU, and RPI for sensor connectivity. More concretely, they first used ZigBee and Node-MCU to collect different sensors data and then send the acquired data to RPI for further processing which takes much time. However, in our proposed solution, we used RPI to connect all the sensors directly which reduces delay in the response time of device switching. The data communication between IoT devices and edge devices is in both modes (short and long-range). If the sensors are connected in the range of Wi-Fi, then the system uses short-range communication, otherwise, it uses the cloud resources for communication. In our proposed scenario, the direct accessibility of different sensor data via RPI makes the system efficient in terms of response time and turning on and off home appliances to reduce electricity consumption.

## 5. Conclusions

In this study, we presented a fully automated IoT-based hierarchical framework for smart homes that takes advantage of edge-computing devices for data processing and storage. Related systems are proposed in the literature, but many of them cannot fully use the IoT-based platform. The proposed framework provided a proof-of-concept design to extend the existing technology to a new level and introduce edge-based multimedia data processing that enabled the low-cost computation to the edge of the IoT network. This not only reduced the computational cost but also reduced the network bandwidth and storage over the costly cloud solution. Through the working prototype, we conclusively proved that the proposed system is much efficient in terms of energy consumption, response time, data processing, and bandwidth use.

Our system uses data gathered from different sensors including camera, temperature, which could be further used to make various types of intelligent predictions to enhance the existing functionality, such as human fall detection, indoor fire and smoke detection, suspicious activity recognition, etc. Furthermore, deep learning models can be employed to learn individuals’ usage patterns of various home devices so the system can provide personalized management of resources. In the future, we are planning to adopt and integrate efficient, deeply embedded vision-enabled technology to the smart home and use the RPI as a processing unit to inexpensively process the multimedia data such as surveillance videos to support enhanced functionality and reduce storage and bandwidth cost even further. Currently, we have not implemented plug-in support to automatically detect new sensors, but such a functionality could be provided in the future, allowing the proposed framework to be easily configurable and generalizable across various environments. The proposed solution not only provides automation to control various home appliances, but it could also serve as a security system with vision sensors, can provide safety and wellbeing of its inhabitants by monitoring fall detection, smoke detection, or file alert, to name just a few. 

## Figures and Tables

**Table 1:** Performance of the proposed system vs. state-of-the-art concerning energy consumption.

City	Model	Energy Consumption Annual (KWH)	Per day an Average Energy Consumption (Watts)
Algiers, Algeria	Baseline model [43]	44,000	5022.83
	Low cost [43]	37,000	4282.40
	Extended [43]	33,000	3819.44
Stuttgart, Germany	Base line model [43]	44,000	5022.83
	Low cost [43]	34,000	3881.27
	Extended [43]	30,000	3424.65
Peshawar, Pakistan	Our manual operated system	39,000	4452.05
	Proposed automatic system	24,001	2739.84

**Table 2:** Performance of the proposed system vs. state-of-the-art concerning response time.

System	Scenario	Response Time
Proposed (RPI-based system)	When motion is detected, turn ON the light	15 ms
	Relay switch ON	13 ms
	Relay switch OFF	12 ms
ZiWi-Fog [54]	Turn on a light when low luminosity is detected	20 ms
	Relay switch ON	18 ms
	Relay switch OFF	16 ms
ZiWi-Cloud [54]	Detected and notify alert through Telegram	2.735 s
	Turn on a light when low luminosity is detected through IFTTT	51.388 s
ZigBee [55]	Relay ON/OFF	2 s

## Data Availability

Not applicable.

## Appendix A

**Table A1:** Summary of different smart home approaches.

Reference	Smart Home Solution	Hardware Deployed	Features	Limitations
[30]	Remote controlling	Arduino and ZigBee	GSM-based system	Only remote controlling of home appliances.
[13]	Remote controlling	Zigbee	Wi-Fi-based communication	Only remote controlling of home appliances based on ZigBee.
[16]	Home automation	Arduino	Temperature, humidity, and current-based system	Only home automation of home appliances.
[14]	Home automation	Arduino	Temperature-based system	Only automation Used Matlab-GUI expensive solution.
[28]	Home automation	Arduino	PIR and temperature-based system	only home automation.
[31]	Home automation	Arduino	Bluetooth-based system	Home automation Works in a very limited geographical area.
[32]	Home automation	Arduino	Wi-Fi, Bluetooth, and Zigbee-based system	3 different communication protocols are used for different technologies such as Wi-Fi, Bluetooth, and Zigbee. Unfeasible and costly in terms of implementation.
[35]	Home security	Arduino and RPI	PIR sensor, Camera, and machine learning-based system	High ratio of misclassification False alarm generation.
[36]	Home security	fog computing technology	analysis of foot pressure, size, and movement in real time to detect personnel identity	Only focused on the security aspect of smart homes using ANFIS.
[41]	Home energy management system	ZigBee	PLC-based energy monitoring system	Only focused on home energy management systems using solar and wind. Used a heavy server.
[29]	Home Energy management	Arduino and ZigBee	RCPSP-based system	Only work on energy management based on priority-based scheduling mechanisms.
Proposed work	Home automation, safety and security, and energy consumption	RPI	Photoresistor, motion detection, fingerprint authentication, water, temperature, camera, flame, and gas sensors-based system and MQTT protocol is used for data transmission. Data storage is different from the rest, we used own storage device at the edge of the home network to reduce dependency on cloud resources	In the proposed work, we used camera only for online streaming, in future we can used it for any anomaly detection in our home, such as wall crossing, or for any safety and security risk analysis such as flame detection and elderly fall detection.
